# Enhancing reinforcement learning models by including direct and indirect pathways improves performance on striatal dependent tasks

**DOI:** 10.1371/journal.pcbi.1011385

**Published:** 2023-08-18

**Authors:** Kim T. Blackwell, Kenji Doya

**Affiliations:** 1 Department of Bioengineering, Volgenau School of Engineering, George Mason University, Fairfax, Virginia, United States of America; 2 Neural Computation Unit, Okinawa Institute of Science and Technology Graduate University, Okinawa, Japan; University of Tokyo: Tokyo Daigaku, JAPAN

## Abstract

A major advance in understanding learning behavior stems from experiments showing that reward learning requires dopamine inputs to striatal neurons and arises from synaptic plasticity of cortico-striatal synapses. Numerous reinforcement learning models mimic this dopamine-dependent synaptic plasticity by using the reward prediction error, which resembles dopamine neuron firing, to learn the best action in response to a set of cues. Though these models can explain many facets of behavior, reproducing some types of goal-directed behavior, such as renewal and reversal, require additional model components. Here we present a reinforcement learning model, TD2Q, which better corresponds to the basal ganglia with two Q matrices, one representing direct pathway neurons (G) and another representing indirect pathway neurons (N). Unlike previous two-Q architectures, a novel and critical aspect of TD2Q is to update the G and N matrices utilizing the temporal difference reward prediction error. A best action is selected for N and G using a softmax with a reward-dependent adaptive exploration parameter, and then differences are resolved using a second selection step applied to the two action probabilities. The model is tested on a range of multi-step tasks including extinction, renewal, discrimination; switching reward probability learning; and sequence learning. Simulations show that TD2Q produces behaviors similar to rodents in choice and sequence learning tasks, and that use of the temporal difference reward prediction error is required to learn multi-step tasks. Blocking the update rule on the N matrix blocks discrimination learning, as observed experimentally. Performance in the sequence learning task is dramatically improved with two matrices. These results suggest that including additional aspects of basal ganglia physiology can improve the performance of reinforcement learning models, better reproduce animal behaviors, and provide insight as to the role of direct- and indirect-pathway striatal neurons.

## Author summary

Humans and animals are exceedingly adept at learning to perform complicated tasks when the only feedback is reward for correct actions. Early phases of learning are characterized by exploration of possible actions, and later phases of learning are characterized by optimizing the action sequence. Experimental evidence suggests that reward is encoded by the dopamine signal, and that dopamine also can influence the degree of exploration. Reinforcement learning algorithms are machine learning algorithms that use the reward signal to determine the value of taking an action. These algorithms have some similarity to information processing by the basal ganglia, and can explain several types of learning behavior. We extend one of these algorithms, Q learning, to increase the similarity to basal ganglia circuitry, and evaluate performance on several learning tasks. We show that by incorporating two opposing basal ganglia pathways, we can improve performance on operant conditioning tasks and a difficult sequence learning task. These results suggest that incorporating additional aspects of brain circuitry could further improve performance of reinforcement learning algorithms.

## Introduction

Reward learning, which explains many types of learning behavior, is controlled by dopamine neurons and the striatum, which integrates excitatory inputs from all of cortex [1,2]. Reward learning stems from synaptic plasticity of cortico-striatal synapses in response to cortical and dopamine inputs [3–5]. Dopamine is an ideal signal for triggering reward-related synaptic plasticity because activity of midbrain dopamine neurons signals the difference between expected and actual rewards [6,7]. Numerous reinforcement learning theories and experiments demonstrate that many aspects of reward learning behavior results from selecting actions that have been reinforced by the reward prediction error [8–11].

State-action value learning is a type of reinforcement learning algorithm [12], whereby an agent learns about the values (i.e., the expected reward) of taking an action given the sensory inputs (the state), and selects the action based on those values. Q learning is state-action value learning combined with a temporal difference learning rule, in which the value of a state-action combination is updated based on the reward plus the difference between expected future rewards and current value of the state [12]. Q learning models can explain many striatal dependent learning behaviors, including discrimination learning and switching reward probability tasks [11,13–16]. Learning the value of state-action combinations may be realized by dopamine-dependent synaptic plasticity of cortico-striatal synapses [3,5,17–19].

Striatal spiny projection neurons (SPN) are subdivided into two subclasses depending on the expression of dopamine receptors and their projections [20]. The dopamine D1 receptor containing SPNs (D1-SPN) disinhibit thalamus by the direct pathway through the internal segment of the globus pallidus (entopeduncular nucleus in rodents) and substantia nigra pars reticulata; the dopamine D2 receptor containing SPNs (D2-SPN) inhibit thalamus by the indirect pathway through the external segment of the globus pallidus. Accordingly, a common theory is that D1-SPNs promote movement, while D2-SPNs inhibit competing actions [21,22]. Not only do these neurons control instrumental behavior differently [8], but the response to dopamine also differs between SPN subtypes. The conjunction of cortical inputs and dopamine inputs produces long term potentiation of synapses to D1-SPNs [18,23–25], increasing the activity of the neurons which promote the rewarded action. In contrast, a decrease in dopamine is required for long term potentiation in D2-SPNs [17,19].

One reinforcement learning model, Opponent Actor Learning or OpAL [26], is an actor-critic model that includes a representation of both classes of SPNs. In OpAL, there are two sets of state-action values, one corresponding to the D1-SPNs and one to the D2-SPNs: values corresponding to the D2-SPNs are updated with the negative reward prediction error. This model can reproduce the effect of dopamine on several behavioral tasks, which supports the idea that improving correspondence to the basal ganglia can improve reinforcement learning models.

One problem with reinforcement learning models is the inability to show renewal of a response after extinction in a different context. An elegant solution to this problem is the state-splitting model [14], which enables the agent to learn new states and recognize when the context of the environment has changed. In addition to reproducing renewal after extinction, this algorithm has the advantage of minimizing storage of unused states.

The research presented here proposes a biologically motivated Q learning model, TD2Q, that combines aspects of state-splitting and OpAL. Similar to state-splitting, the agent learns new states when the likelihood is low of being in an existing state. Similar to OpAL, the agent has two value matrices: G and N, corresponding to D1-SPNs and D2-SPNs, each of which can have a different set of states. Simulations of two classes of multi-state operant tasks show that this TD2Q model exhibits better performance, similar to that seen in animal learning, compared to a single Q matrix model.

## Methods

### TD2Q learning model

We created a new reinforcement learning model, TD2Q, by combining aspects of the actor-critic model, OpAL [26], and the Q learning model with state-splitting, TDRLXT [14]. As in TDRLXT, the environment and agent are distinct entities, with the environment comprising the state transition matrix, T, and reward matrix, Ψ, and the agent implementing the temporal difference algorithm to learn the best action for a given state. Basically, at each time step, the agent identifies which state it is in (state classification), selects an action in that state (action selection), and then updates the value of state-actions using a temporal difference rule (learning). Following each agent action, the environment determines the reward and next state from the agent’s action, *a*, using the state transition matrix and the reward matrix, and then provides that information to the agent.

The information that defines the dynamics of the environment at time *t* is a multi-dimensional vector of task state, *tsk(t)*, along with the agent’s action, *a*. Both the transition matrix, *T(tsk(t+1)|tsk(t)*,*a)*, and the reward matrix, Ψ*(rwd|tsk(t)*, *a)*, depend on the task state at time *t* and the agent’s action, *a*. The information, *cues(t)*, that is input to the agent at time *t* is an extended multi-dimensional vector comprised of the task state (the output of the environment) together with context cues, *cxt(t)*:

cues(t)=(tsk(t),cxt(t)) (1)

Context cues represent other sensory inputs (e.g. a different operant chamber) or internal agent states (e.g., mean reward over past few trials) that may indicate the possibility of different contingencies.

### Temporal difference learning

The state-action values are stored by the agent in two Q matrices, called G (corresponding to Go, by the direct pathway SPNs) and N (corresponding to NoGo, by the indirect pathway SPNs), following the terminology of [26]. Each row in each matrix corresponds to a single state, s_G_ for states for the G matrix and s_N_ for states for the N matrix, where s_G_ or s_N_ is the state determined by the agent using the state classification step described below. State-action values in both matrices are updated using the temporal difference reward prediction error (δ), which is calculated using the G matrix:

$$\delta (t)=rwd(t)+\gamma \;\underset{a}{max}\{G(s_{G}(t),a)\}-G(s_{G}(t-1),a(t-1))$$ (2)

where γ is the discount parameter, and *s*_*G*_*(t-1)* is the previous state. G values are updated using the temporal difference reward prediction error, δ:

$$G(s_{G}(t-1),a(t-1))=G(s_{G}(t-1),a(t-1))+\alpha_{G}\delta (t)$$ (3)

where *α*_*G*_ is the learning rate for the G matrix.

The N values of the previous action are *decreased* by positive δ (as in [26]), because high dopamine produces LTD in indirect pathway neurons [17,19,27,28].

$$N(s_{N}(t-1),a(t-1))=N(s_{N}(t-1),a(t-1))-\alpha_{N}\delta (t)$$ (4)

where *s*_*N*_*(t-1)* is the previous state corresponding to the N matrix, α_N_ is the learning rate for the N matrix, and δ is the temporal difference reward prediction error defined in Eq (2). More negative values of the N matrix correspond to less indirect pathway activity and less inhibition of motor action. The same value of δ is used for both G and N updates because the dopamine signal is spatially diffuse [29,30]; thus D1-SPNs and D2-SPNs experience similar reward prediction errors. Furthermore, recent research reveals that D1-SPNs in the striosomes (a sub-compartment of the striatum containing both D1- and D2-SPNs) directly project to the dopamine neurons of SNc, which project back to the striatum. Thus, only the D1-SPNs directly influence dopamine release [31–33].

### State-classification and state-splitting

From the task state provided by the environment, together with the additional context cues, the agent determines its state using a simple classification algorithm. Since each matrix of state-action values can have a different number of states, the state is selected for each matrix (G or N) from the set of cues by calculating the distance to all ideal states, *M*_*k*_, *k* ∈ *{G*,*N}*, and selecting the state with the smallest Euclidean distance:

$$\Delta C_{ki}=\sqrt{\Sigma_{j}{w_{j}(c_{j}(t)-M_{kij})}^{2}}$$ (5)

where *c(t) = cues(t) + G(0*,*σ)*, and *G(0*,*σ)* is a vector of Gaussian noise with standard deviation, σ, and *j* is the index into the multi-dimensional cue vector. *M*_*ki*_ is the ideal for state *s*_*ki*_, where *k* ∈ *{G*, *N}*, and *i* ranges from 0 to the number of states, *m*_*k*_. *M*_*ki*_ is calculated as the mean of the set of past input cues, e.g. *c(t)*, *c(t-2)*, *…*, *c(t-trials)* that matched *M*_*ki*_. *w*_*j*_ is a normalization factor, and is the inverse of standard deviation of the cue value for the *jth* index, for those cue indices that have units, e.g. tone cues as explained below. Note that the noise, *G(0*,*σ)*, incorporates uncertainty as to the agent’s observations (e.g., sensory variation due to noise in the nervous system), and thus is added to agent state inputs, but not to environmental states. The best matching state is that state with the smallest distance to the noisy cues:

$${\hat{s}}_{k}(t)=s_{kb},where\;b=arg\underset{i}{min}\Delta C_{ki}$$ (6)

where *k* ∈ *{G*,*N}*, ${\hat{s}}_{G}(t)$ and ${\hat{s}}_{N}(t)$ are the best matching state at time *t* for G and N, respectively. ${\hat{s}}_{k}(t)$ is selected as the new state, providing that

$$\underset{i}{min}\Delta C_{ki}<{ST}_{k}$$ (7)

where *ST*_*k*_, is the state creation threshold. Otherwise, a new state is created with *M*_*ki*_ *= c(t)*, *i = m*_*k*_ and *m*_*k*_ is incremented by 1. In other words, the state vector of the new state is initialized to the current set of input cues. Each time a best matching state is selected, *M*_*ki*_ is updated once the number of observations (set of past input cues) of state *i* exceeds the state history length. When a new state is created, the row in the matrix (G or N) for the new state is initialized to the G or N values of the best matching state (that with the highest probability), a process called *state-splitting*:

$$Q_{k}[m_{k}]=Q_{k}[\hat{s_{k}}]$$ (8)

where Q_k_ is either G or N. Since the state threshold and learning rates differ for G and N, the number of rows (and ideal states) may differ. There are three differences between TDRLXT [14] and TD2Q: **1.** Eqs 5–8 are applied to both G and N, instead of a single Q matrix, **2.** Values of the new states are not initialized to 0 or a positive constant, but instead initialized to the values of the most similar state, and **3.** The weights are not calculated from mutual information of the cue. In addition, a Euclidean distance is utilized instead of Mahalanobis distance to determine whether a new state is needed. However, similar results are obtained when the Mahalanobis distance is used, though different state thresholds are required.

### Action selection

The agent’s states (one for G and one for N) are used to determine the best action in a two step process. First, the softmax equation is applied to single rows in both G and N matrices to select two best actions:

$$P_{k}=P(a_{k}|s_{k}(t))=\frac{exp(\beta_{1}Q_{k}(\hat{s_{k}}(t),a))}{\underset{a}{\Sigma }exp(\beta_{1}Q_{k}(\hat{s_{k}}(t),a))}$$ (9)

where β_1_ is a parameter the controls exploration versus exploitation, *k* ∈ *{G,N}*, ${\hat{s}}_{k}(t)$ are the best matching states for G and N, and Q_k_ is either G or N. Note that negative values are used in Eq 9 when selecting the best action from the N matrix, to translate more negative N values into more likely actions, reflecting that lower N values implies less inhibition of basal ganglia output (motor activity). Two actions, a_k_′, are randomly selected from distributions P_k_, *k* ∈ *{G*,*N}*.

Second, if the actions, *a*_*k*_′ selected using G and N, disagree, the agent’s action, *a*, is determined using a second softmax applied to the probabilities, *P*_*k*_′ = [*P*_*G*_′, *P*_*N*_′], corresponding to the actions, *a*_*k*_′, determined from the first softmax:

$$P(a_{k}^{\prime }|{P_{k}}^{\prime })=\frac{exp(\beta_{2}{P_{k}}^{\prime })}{\underset{f}{\Sigma }exp(\beta_{2}{P_{k}}^{\prime })}$$ (10)

where k ∈ *{G*,*N}*, *β*_*2*_ is a second parameter the controls exploration versus exploitation for this second level of action selection.

To mimic another role of dopamine [34–37], the exploitation-exploration parameter β_1_ is adjusted between a user specified minimum, β_min_, and maximum, β_max_, based on the mean reward:

$$\beta_{1}=\beta_{min}+(\beta_{max}-\beta_{min})*\bar{rwd}$$ (11)

where $\bar{rwd}$ is the running average of reward probability over a small number of trials (e.g. 3, [38]). The number of trials can be greater, especially if the task is more stochastic or with fewer switches in reward probability, but 3 works well for the tasks considered here, as in [38].

### Tasks

We tested the TD2Q model on several operant conditioning tasks, each selected to illustrate the role of one or more features of the model. One set of tasks investigated the agent’s basic ability to associate a cue with an action that yields reward. This set of tasks included extinction and renewal, which are used to investigate relapse after withdrawal in drug addiction research [39,40]; discrimination learning, which requires synaptic potentiation in D2-SPNs [19]; and reversal learning, which tests behavioral flexibility [41,42]. The second task was switching reward probability learning [11,16,43], in which two actions are rewarded with different probabilities. As the probabilities can change during the task, the agent must continually learn which action provides the optimal reward. The third task was a sequence learning task [44], which requires the agent to remember the history of its lever presses to make the correct action. To better compare with animal behavior, for each of these tasks, a single trial requires several actions by the agent, and the agent’s action repertoire included irrelevant actions often performed by rodents during learning.

**Fig 1:**
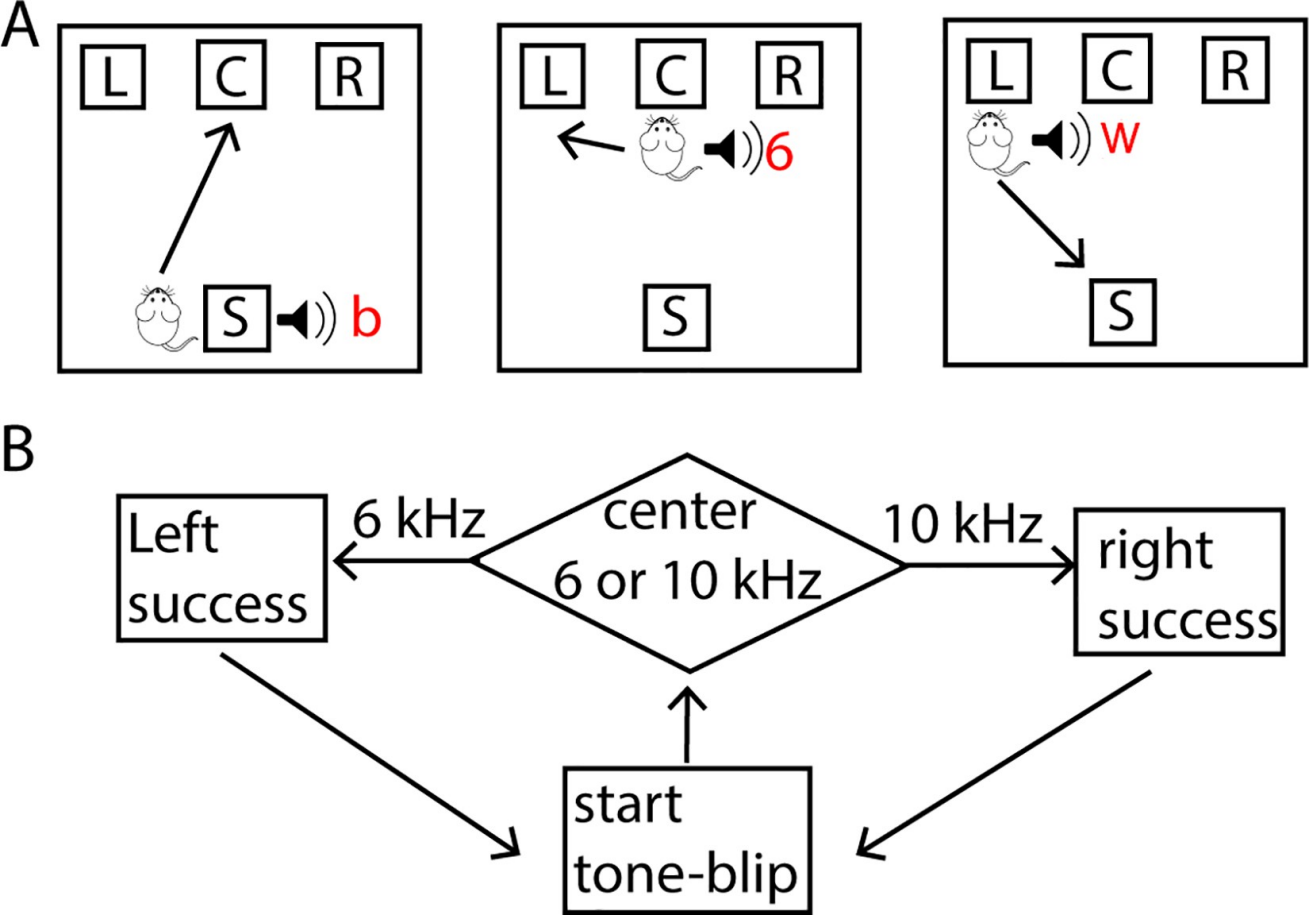
Optimal acquisition and discrimination sequences. The environment input is a 2-vector of (location, tone). Location is one of: *start location*, *left poke port*, *right poke port*, *center poke port*, *food magazine*, *other*. Tone is one of: *start tone*, *success tone*, *6 kHz*, *error* and (during discrimination and reversal) *10 kHz*. The agent input is a 3-vector of (*location*, *tone*, *context*), where location and tone are the same as the environment input, and context is either A or B. Possible actions included: *return to start*, *go to left port*, *go to right port*, *go to center port*, *hold*, *groom*, *wander*, *other*. **A.** Sequence of state-actions to maximize reward during acquisition of left poke in response to 6 kHz tone. At (*start location*, *tone blip*), go to center poke port. At (*center port*, *6 kHz tone*), go to left poke port. At (*left poke port*, *success tone*), return to start. A reward was provided on 90% of (*left poke port*, *success tone*) trials. C: center, L: left, R: right, S: start, b: blip, 6: 6 kHz tone, w: reward. In response to action *wander* the agent location is other parts of the chamber. Neither *hold* nor *groom* changes the location of the agent, but these actions reduce reward, as they lengthen the number of actions needed to obtain reward. **B.** During the discrimination task, either the 6 kHz or 10 kHz tone occurs with 50% probability. The agent is required go *left* in response to the 6 kHz tone and *right* in response to the 10 kHz tone to receive a reward.

The first set of tasks was simulated as one of two sequences of tasks: either acquisition, extinction, renewal; or acquisition, discrimination, reversal. During acquisition, the agent learns to poke *left* in response to a *6 kHz* tone to receive a reward over the course of 200 trials, as in [19]. Fig 1A shows the optimal sequence of three actions during acquisition: go to the center port in response to start cue, poke *left* in response to *6 kHz* tone, and then return to the start location to collect reward and begin next trial. To test extinction, acquisition occurred with context cue A, and then context cue B was used while the agent experienced the same tones but did not receive a reward. To test renewal, after extinction with context cue B, the agent was returned to context A and again the agent experienced the same tones but did not receive a reward. To test discrimination, the agent first acquired the single tone task, and then a second tone, requiring a right turn for reward, was added (Fig 1B). During the 200 discrimination trials, *6 kHz* and *10 kHz* tones each occur with 50% probability in the poke port. To test reversal learning after the discrimination trials, the tone-direction contingency was switched; thus, the agent had to learn to go *right* after a *6 kHz* tone and *left* after the *10 kHz* tone. The possible actions, as well as location and tone inputs are listed in Table 1.

**Table 1:** Actions and states (location and tone) for the discrimination and switching reward probability tasks. Note that “groom” and “other” actions were not available in the switching reward probability task.

Action	Action code	Location state	Location code	Tone state	Tone code
go to center port	0	start/food magazine	0	Start cue	0
go left	1	Left port	1	Success	2
return (to start/food magazine)	2	center port	2	6 kHz	6
go right	3	Right port	3	10 kHz	10
wander	4	Other	4	error	-2
hold	5				
groom	6				
other	7				

For the switching reward probability task, from (*start location*, *tone blip*) the agent must go to the center poke port. At the center port, the agent hears a single tone (go cue) which contains no information about which port is rewarded. To receive a reward, the agent has to select either left port or right port. Both left and right choices are rewarded with probabilities assigned independently. The pairs of probabilities are listed in Table 2. After selecting left or right port, the agent must return to the start location for the next trial.

**Table 2:** Pairs of reward probabilities for the switching reward probability task. The order of the pairs was randomized for each agent. Probabilities are expressed as percent.

Left reward probability	Right reward probability
10%	90%
10%	50%
50%	90%
50%	50%
50%	10%
90%	50%
90%	10%

In the sequence learning task [44], the agent must press the left lever twice and then the right lever twice to obtain a reward. There are no external cues to indicate when the left lever or right lever needs to be pressed. Both environment and agent inputs are a 2-vector of *(location*, *press sequence)*. The location is one of: *left lever*, *right lever*, *food magazine*, *other*. The press history is a string containing the history of lever presses, e.g. ‘LLRR’, ‘LRLR’, etc, where L indicates a press on the left lever and R indicates a press on the right lever. The most recent lever press is the right most symbol. New lever presses shift the press history sequence to the left, with the oldest press being removed from the press history. Possible actions include: *go to right lever*, *go to left lever*, *go to food magazine*, *press*, *other*. The agent is rewarded for going to the food port when lever press history is ‘LLRR’. Table 3 illustrates the action sequence and resulting states for optimal rewards.

**Table 3:** State, action sequence for optimal rewards. At the start of the task and after a reward, the press sequence is initialized to empty (----) and the agent location is *food magazine*. R indicates a right lever press and L indicates a left lever press in the press history.

State (location, press history)	Best action	Reward
(food port, ----)	Go to left lever	-1
(Left lever, ----)	Press	-1
(Left lever, ---L)	Press	-1
(Left lever, --LL)	Go to right lever	-1
(Right lever, --LL)	Press	-1
(Right lever, -LLR)	Press	-1
(Right lever, LLRR)	Go to food port	15

The acquisition-discrimination and sequence tasks were repeated using a range of learning rates, encompassing the rates in other published models [14,26,45,46] (α_1_ = [0.2,0.3,0.4,0.5,0.6,0.7,0.8], α_2_ = [0.1,0.15, 0.2,0.25,0.3,0.35,0.4]) and state thresholds (ST_1_, ST_2_ = [0.5,0.625,0.75,0.875,1.0]), for both one and two matrix versions of the discrimination and sequence tasks. Optimal parameters were those producing the highest reward at the end in the sequence task, and the highest acquisition and discrimination reward for the discrimination task. Using those parameters, simulations were repeated 10 times for the discrimination and extinction task, and 15 times for the sequence task. The switching reward probability task was simulated 40 times using the state threshold parameters determined for the discrimination task, and learning rates two fold higher than used in the discrimination task, so that agents could learn the task within the number of trials used in rodent experiments [43]. Using these optimal parameters, we investigated the effect of γ and β_max_ using a range of values encompassing previously published values. β_min_ ranged from the lowest β_max_ down to a value to show a decrement in performance. Table 4 summarizes the parameters used for the three tasks.

**Table 4:** Parameters.

Parameter	Extinction, discrimination, reversal	Switching reward probability	Sequence
learning rate: α or [α_G_, α_N_],	Q: 0.3; G,N: [0.2,0.1]	Q: 0.6; G,N: [0.4,0.2]	Q: 0.2; G,N: [0.2,0.35]
β_min_, β_max_	0.5, 1.5	0.5, 1.5	0.5, 3
β_2_	10	10	10
γ, discount factor	0.82	0.82	0.95
state threshold: [ST_G_,ST_G_]	[0.75,0.625]	[0.75, 0.625]	[0.5,0.875]
σ, noise	0.15	0.15	0.01
State history length	40	40	40
Minimum actions to reward	3	3	7
Number of actions per run	600 for each phase	300 for each probability pair	4200
Moving average window for reward probability	3	3	3

All code was written in python3 and is freely available on github (https://www.github.com/neuroRD/TD2Q). Graphs were generated using the python package matplotlib or IgorPro v8 (WaveMetrics, Lake Oswego, OR), and the difference between one Q matrix versus G and N matrices was assessed using a ttest (scipy.ttest_ind). Each task was run for a fixed number of actions by the agent, analogous to fixed time sessions used in some rodent experiments. One trial is defined as the minimum number of actions required to obtain a reward. Reward rate and response rate per trial are calculated using this minimum (optimal) number of actions for each task. Thus, if an agent performs additional actions (e.g. groom or wander in the discrimination task or additional lever presses in the sequence task), the response rate is less than 1. This is analogous to a rodent taking more time to complete a trial and thus completing fewer trials and receiving fewer rewards per unit time.

## Results

We tested the TD2Q reinforcement learning model on several striatal dependent tasks [11,16,19,42–44,47,48]. In all tasks, the agent had a small number of locations it could visit (Fig 1A), and the agent needed to take a sequence of actions to obtain a reward.

### Operant conditioning tasks

The first set of tasks tested acquisition, extinction, renewal; or acquisition, discrimination, reversal, and they were simulated as operant tasks, not classical conditioning tasks. Renewal, also known as reinstatement, refers to performing an operant response in the original context after undergoing extinction training in a different context. Mechanisms underlying renewal are of interest because renewal is a pre-clinical model of reinstatement of drug and alcohol abuse [39,40]. Reversal learning tests behavioral flexibility of the agent in the face of changing reward contingencies, and is impaired with lesions of dorsomedial striatum [41,42,49].

**Fig 2:**
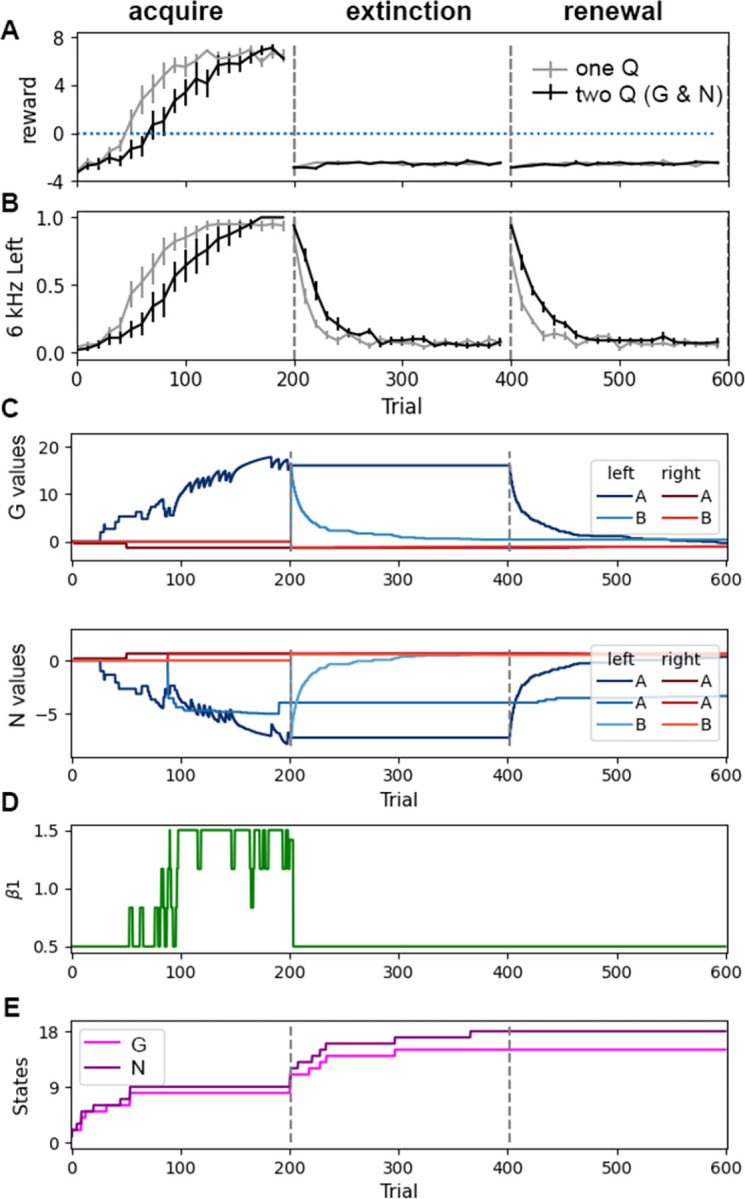
Performance on acquisition, extinction and renewal is similar for one and two matrices. **A.** Mean reward per trial. Agent reaches asymptotic reward within ~100 trials. The difference between agents with one Q versus G and N on the last 20 trials is not statistically significant (T = -0.173, P = 0.866, N = 10 each). **B.** Left responses to 6 kHz tone per trial, normalized to optimal rate during acquisition. Both agents extinguish similarly in a different context (Context B). When returned to the original context (Context A), both agents exhibit renewal: they initially respond as if they had not extinguished; thus, the agents poke *left* in response to 6 kHz tone during first few blocks of 10 trials. **C.** Dynamics of G and N values for state *(Poke port*, *6 kHz)* for a single agent. **D.** β_1_ changes according to recent reward history; thus, β_1_ increases during acquisition, and then remains at the minimum during extinction and renewal. **E.** Number of states of G and N matrices for a single agent. In all panels, gray dashed lines show boundaries between tasks.

In *acquisition*, *extinction and renewal*, the task starts in context A where *left* in response to *6kHz* is rewarded, then (extinction) switches to context B where the same action is not rewarded, and finally (renewal) is returned back to the original context A but *left* is not rewarded. Fig 2A shows the mean reward per trial and Fig 2B shows *left* responses per trial to the *6 kHz* tone. The trajectories and final performance values are quite similar whether one or two Q matrices are used. The trajectories during acquisition and extinction are similar to that observed in appetitive conditioning experiments [19,50,51]. During extinction, the agent slowly decreases the number of responses, requiring about 20 trials to reduce responding by half, as observed experimentally. Fig 2B shows that after extinction of the response (in the novel context, B), the agent continues to go *left* in response to *6 kHz* during the first few blocks of 10 trials when returned to the original context, A. This behavior, replicating what is observed experimentally [50], is explained by the change in state-action values during these tasks (Fig 2C): at the beginning of extinction in context B, the G and N values for *left* in response to *6kHz* are duplicated for the new state with context B by splitting from the G and N values with context A, and then extinguish. Consequently, the number of states for both G and N increase when the agent is placed in the context B (Fig 2E). When the agent is returned to context A, the G and N values corresponding to *left* in response to *6kHz* in context A decrease in absolute value. Fig 2D shows that the value of β_1_ increases (toward exploitation) as the agent learns the task, and then decreases sharply to the minimum during the extinction and renewal tasks due to lack of reward.

**Fig 3:**
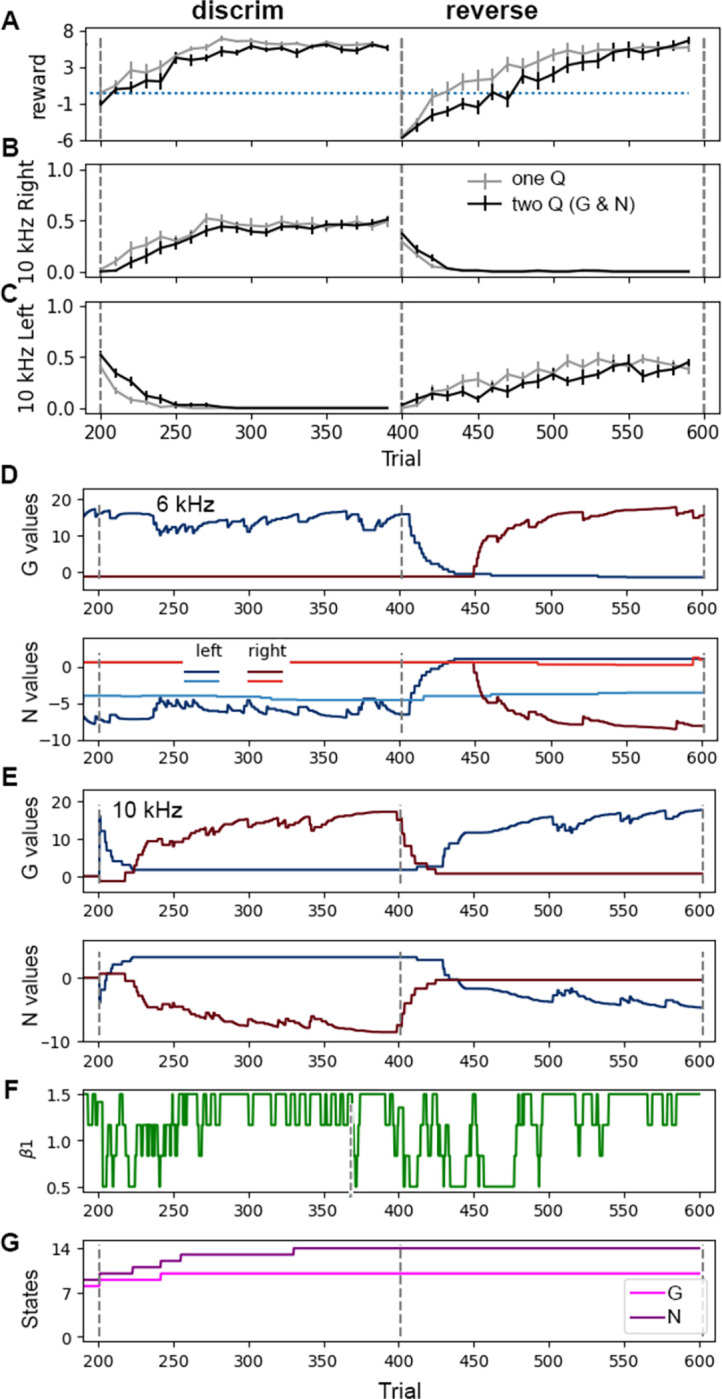
Performance on discrimination and reversal tasks are similar for agents with one Q versus G and N matrices. After acquisition (i.e., at trial 200), a second tone is added and the agent must learn to poke right in response to 10 kHz tone. Then, the pairing is switched and the agent learns poke *right* in response to 6 kHz and poke *left* in response to 10 kHz. **A.** Mean reward per trial. The reward obtained during the last 20 trials does not differ between agents with one Q versus G and N matrices for discrimination (T = 0.121, P = 0.905, N = 10 each) or reversal (T = -1.194, P = 0.250, N = 10 each). **(B&C)** Fraction of responses per trial; optimal would be 0.5 responses per trial, since each tone is presented 50% of the time. **B.** During 1^st^ few blocks of discrimination trials, agent goes *Left* in response to 10 kHz tone, exhibiting generalization. **C.** After the first few blocks, the agent learns to go *Right* in response to 10 kHz. After reversal, the agent suppresses right response to 10 kHz. **D.** Dynamics of Q values for state *(Poke port*, *6 kHz)* for a single run with G and N matrices. Note that two different states (rows in the matrix) were created in the N matrix for this agent. **E.** Dynamics of G and N values for state *(Poke port*, *10 kHz)* for a single run. **F.** Dynamics of β_1_ change according to recent reward history; thus, β_1_ decreases at the beginning of discrimination, increases as the animal acquires the correct *right* response, and then decreases to the minimum at reversal. **G.** number of states of G and N matrices for a single run. In all panels, gray dashed lines show boundaries between tasks.

In the *acquisition*, *discrimination and reversal* task, the agent was first trained in the single tone task, and then a second tone of 10 kHz, requiring a *right* response for reward, was added. This is similar to the tone discrimination task used to test the role of D2-SPNs [19,52]. After the discrimination trials, the actions required for the 6 kHz and 10 kHz tones were reversed, to test reversal learning. Fig 3 shows that performance on the discrimination and reversal task are similar whether one Q or G and N matrices are used, and the trajectories are similar to experimental discrimination learning tasks [19]. The agent initially pokes *left* in response to *10 kHz*, generalizing the concept of *left* in response to a tone (Fig 3C). After 30–60 trials, the agent learned to discriminate the two tones and to poke *right* in response to *10 kHz* (Fig 3B). After the reversal (trial 400), both agents reverse over 20–80 trials. When the 10 kHz tone is introduced, new states are created (Fig 3G) and generalization occurs because the G and N values for *10 kHz* (Fig 3E) are inherited (via state-splitting) from the G and N values, respectively, for *6 kHz* (Fig 3D). With continued trials, the G value for *10 kHz*, *left* decreases and the G value for *10 kHz*, *right* increases. During the reversal, the G and N values for *6 kHz*, *left* and *10 kHz*, *right* decay toward zero, and only then do the values for *10 kHz*, *left* and *6 kHz*, *right* increase. β_1_ decreases at the beginning of discrimination (Fig 3F) and then increases once the agent begins turning right. β_1_ decreases again at reversal, which facilitates the agent exploring alternative responses.

**Fig 4:**
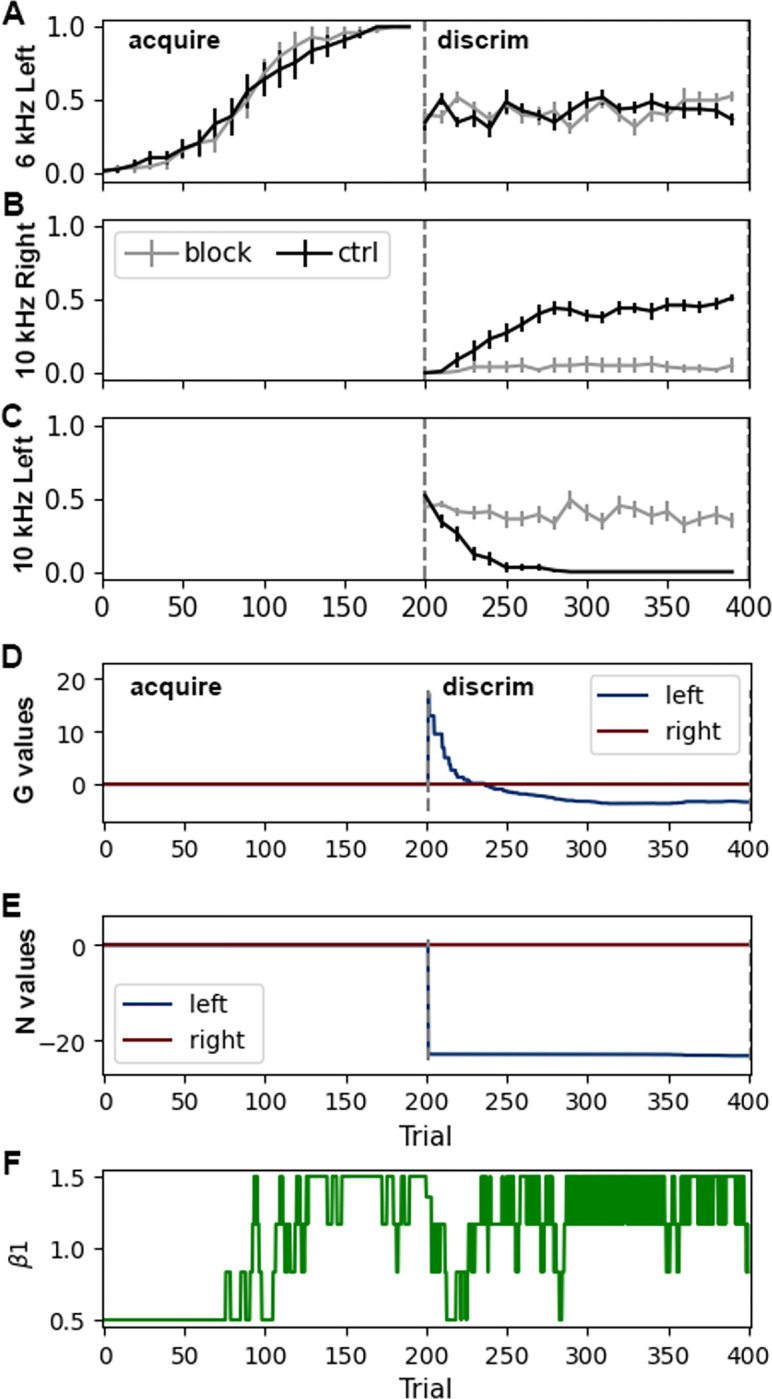
Preventing value increases for the N matrix (analogous to blocking LTP in D2-SPNs) hinders discrimination learning. **A.** Acquisition is not impaired by preventing N value increases. **B**. Agent does not learn to respond *right* to 10 kHz tone when increases in N values are blocked. **C**. Agent continues to respond *left* to 10 kHz tone. Panels A through C show number of responses per trial. **D.** G value for *left* in response to *(Poke port*, *10 kHz)* are defined due to state splitting, and then decrease. **E.** N value for *left* in response to *(Poke port*, *10 kHz)* decreases sharply due to state splitting, and does not increase toward zero because LTP is blocked. **F.** Change in β_1_ values shows an increase as the agent acquires the task and then decreases when discrimination begins. β_1_ is calculated from Eq 11 using the mean reward on the prior 3 trials.

To test the hypothesis that appropriate update of the N matrix is needed for discrimination, we implemented a protocol similar to blocking LTP in D2-SPNs using the inhibitory peptide AIP [19], which blocks calcium-calmodulin-dependent kinase type 2 (CamKII). We blocked increases in the values of the N matrix (corresponding to LTP), but allowed decreases in N values (corresponding to CamKII-independent LTD). The agent was trained in acquisition followed by discrimination under these conditions. Fig 4 shows that the agent had no acquisition deficit, but was unable to learn the discrimination task, as observed experimentally [19]. During the discrimination phase, the agent continues to go *left* in response to *10 kHz* (Fig 4C) and does not learn to discriminate the two tones. The G and N values for *left* in response to *10 kHz* split from the G and N values for *left* in response to *6 kHz* (Fig 4D and 4E). With subsequent trials, the G value decreases, but the N value remains strongly negative (Fig 4E), which prevents the agent from choosing *right*. β_1_ dips briefly at the beginning of discrimination and then remains moderately high because the agent is rewarded on half the trials.

**Fig 5:**
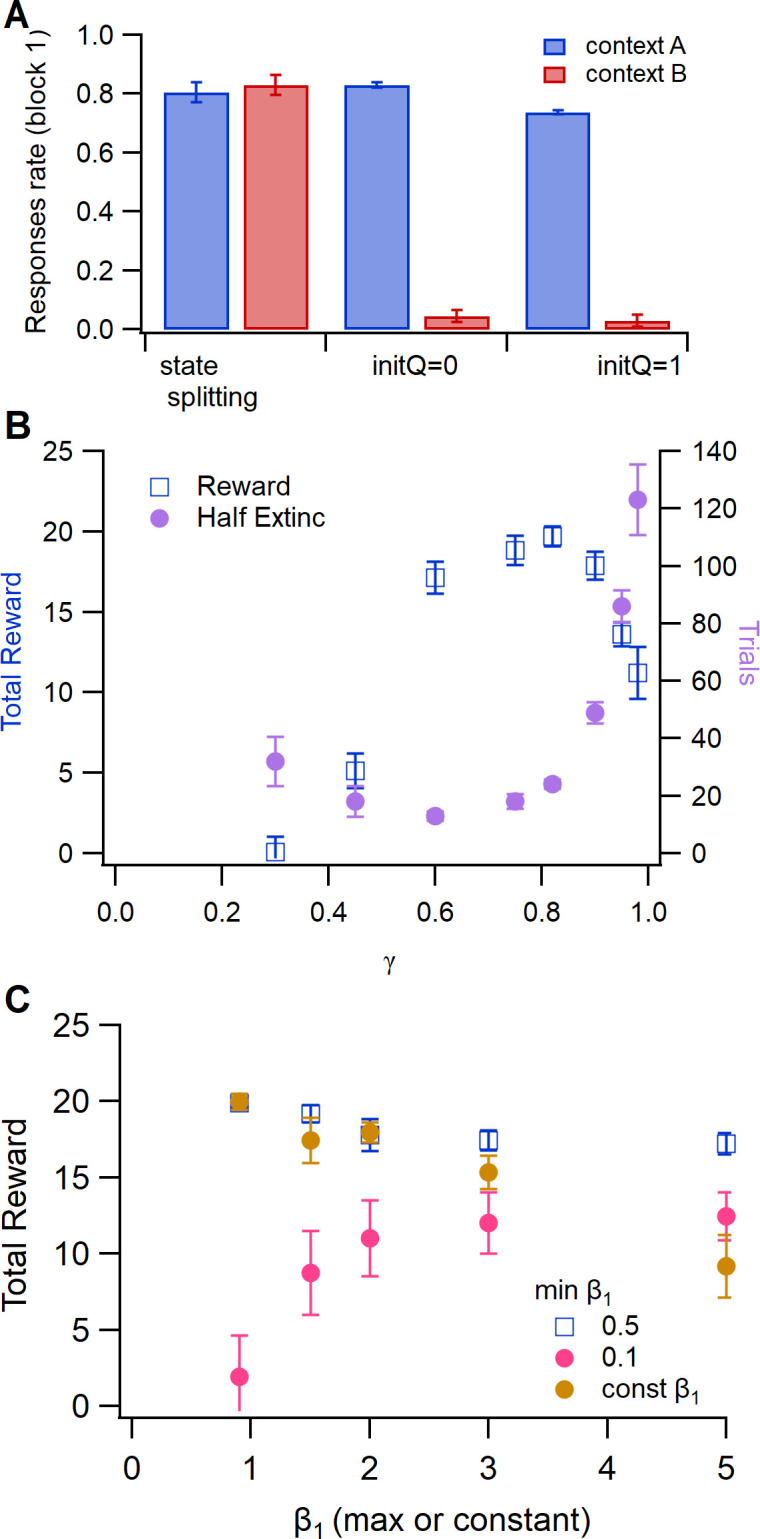
State splitting, γ and exploitation-exploration parameter β_1_ influence specific aspects of task performance. **A.** Number of *Left* responses to 6 kHz tone per 10 trials during extinction in context B (extinction) and context A (renewal). In the absence of state splitting, agent does not respond in the novel context. **B.** Total reward (reward per trial summed over acquisition, discrimination and reversal) and extinction (number of trials until response rate drops below 50%) are both sensitive to γ. Total reward varies little with γ between 0.6 and 0.9; however the rate of extinction is highly sensitive to γ. **C.** Total reward has very low sensitivity to minimum and maximum value of β_1_, unless the minimum is quite small (e.g. 0.1) or maximum quite large (e.g. 5).

Fig 5 illustrates which features are required for the task performance. State-splitting was essential, as eliminating it prevented the agent from exhibiting the correct behavior during extinction and renewal. Specifically, during the extinction phase, the agent recognizes that the context is different and a new state is created, but without state-splitting, this new state is initialized with values = 0, and thus the agent does not press the left lever (Fig 5A). Initializing G and N values to 1.0 instead of zero does not allow the agent to respond in the novel context. Using both G and N matrices is not essential for this task, as shown in Figs 2 and 3; however, the N matrix was essential to reproduce the experimental observation that blocking D2 receptors (value update of the N matrix) impairs discrimination learning [25].

Using the temporal difference rule is critical for this task, as reducing γ toward 0 dramatically impairs performance on these tasks. The value of γ determines how much future expected reward influences the change in state-action values and thus is essential for this multi-step task. Fig 5B shows that γ influences both reward per trial and extinction rate. If values are 0.9 or greater, extinction is delayed and does not match rodent behavior. Within the range of 0.6–0.9, the reward (summed over acquisition, discrimination and reversal) is robust to variation in the value of γ, thus we selected γ = 0.82 to match experimental extinction rates.

Modulating the exploration-exploitation parameter, β_1_, is not critical, but can influence the reward rate if values are too high or too low. Fig 5C shows that performance declines using a constant β_1_ if β_1_ is too high. Using a reward driven β_1_ makes performance less sensitive to the limits placed on β_1_, even though low values of β_min_ and β_max_ prevent the agent from sufficiently exploiting once it learned the correct response. In contrast, values of β_1_ have very little influence on the rate of extinction in this task.

### Switching reward probability learning

We implemented a switching reward probability learning task [11,16,43] to test the ability of the agent to learn which action produces higher rewards under changing reward contingencies and when rewards are only available in part of the trials. In this task, the agent can choose to go *left* or *right* in response to *6 kHz* tone at the center port. Both responses are rewarded, though with different probabilities that change multiple times within a session. This task requires the agent to balance exploitation–choosing the current best option–with exploration–testing the alternative option to determine if that option recently improved.

**Fig 6:**
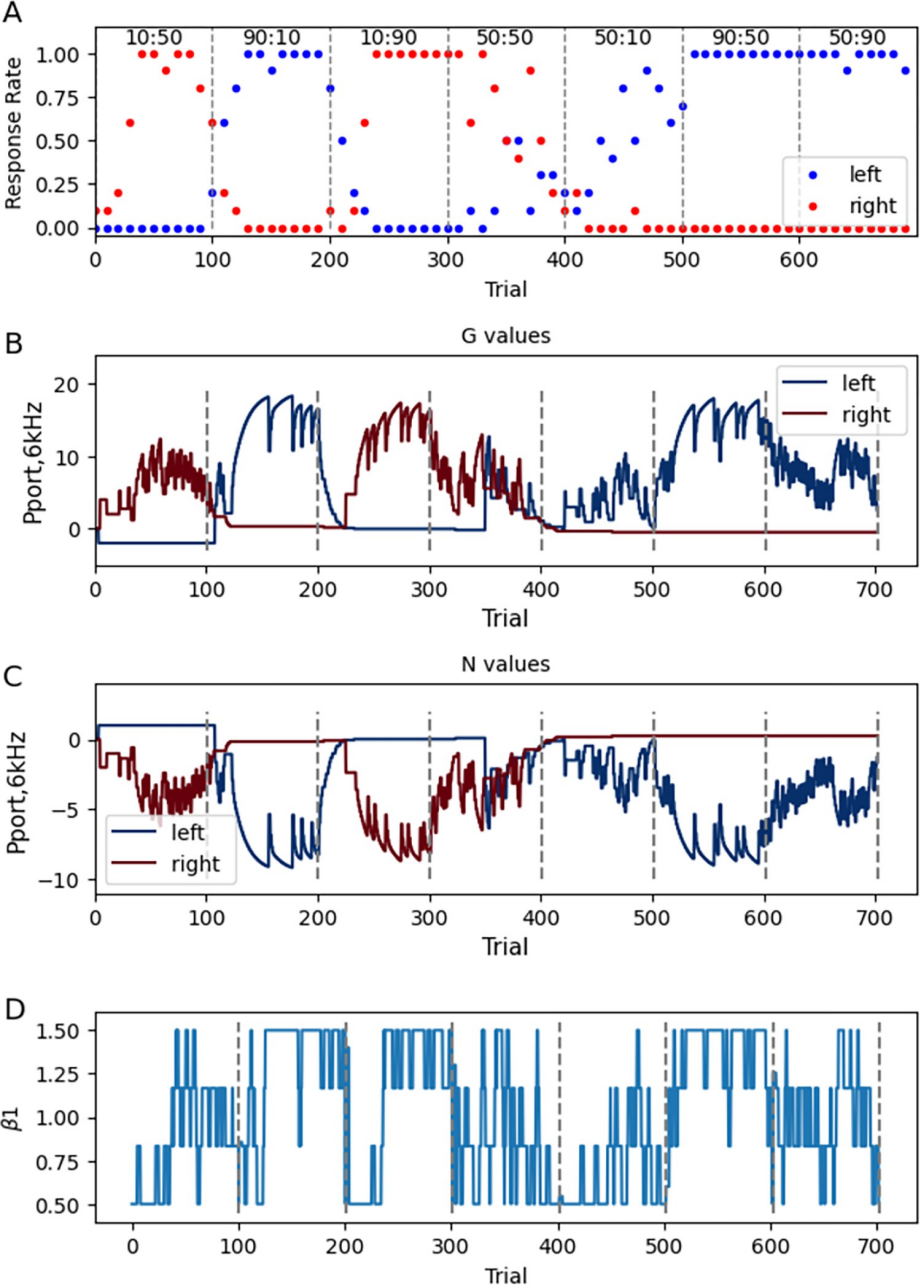
Example of responses and G and N values for single agent in the switching reward probability task. **A.** Number of left and right responses per trial. **B.** G values, and **C.** N values for left and right actions in response to 6 kHz tone in the poke port. **D.** Change in β_1_ values for single agent. β_1_ decreases when reward probability drops.

Fig 6 shows the behavior of one agent (Fig 6A) and the accompanying G and N values (Fig 6B and 6C) for *left* and *right* actions in response to *6 kHz* at the center port. When one of the reward probabilities is 0.9, once the agent has discovered the high probability action, it rarely samples the low probability action. The agent is more likely to try both left and right actions when the reward probabilities are similar for both actions. Note that when left and right response probabilities sum to less than 1, the agent is trying other actions, such as wander or hold, and thus is performing less than optimal. The delay in switching behavior from *left* to *right* when the left reward probability changes from 90% to 10%, e.g. at trial 200, is similar to that observed experimentally, e.g. Fig 1 of [13], Fig 1 of [43]. Note that the G and N values do not reach stable values within a session. The change in values reflect the changing reward probabilities, and the lack of a steady state value also is caused by using only 100 trials per probability pair, to match experimental protocols.

**Fig 7:**
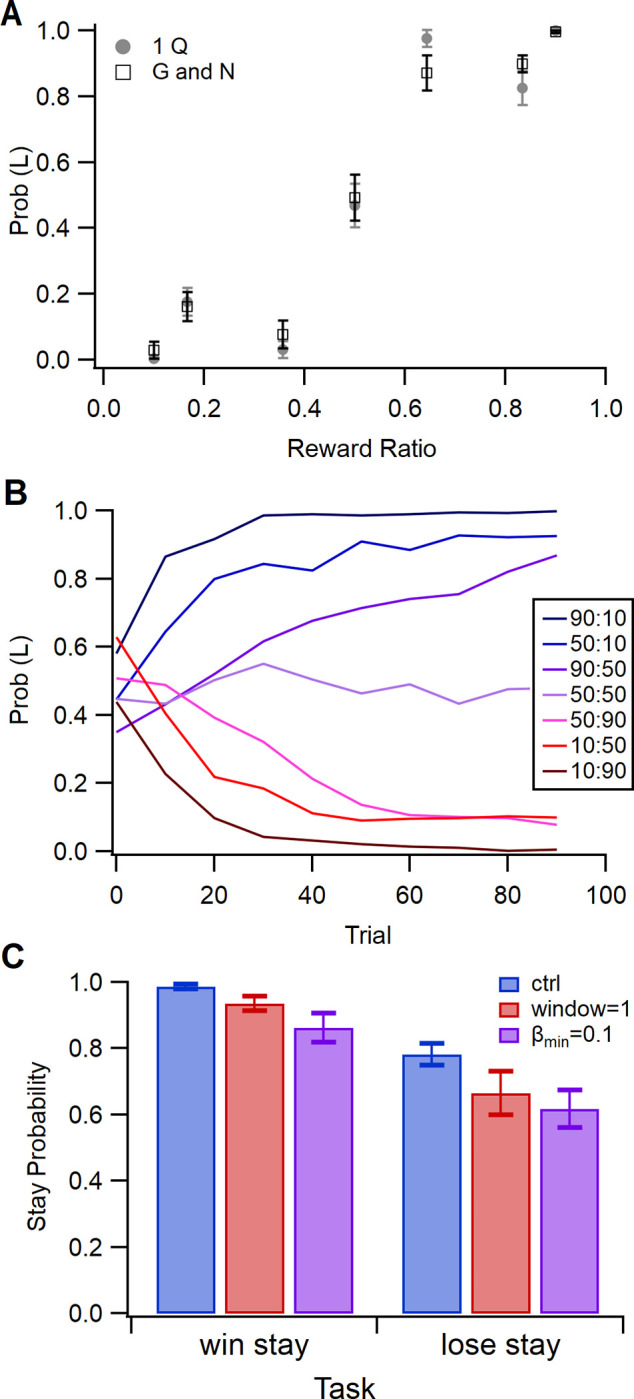
The probability of the agent choosing *left* for each probability pair in the switching reward probability task. **A.** Probability of choosing *left*, by agent with G and N matrices. **B.** Probability of choosing *left* versus reward ratio, p(reward | L) / (p(reward | L) + p(reward | R)), is similar for one Q and two Q agents. **C.** Probability of repeating a response following a rewarded trial (win) and non-rewarded trial (lose) when both *left* and *right* are rewarded half the time. β_min_ = 0.1 decreases the probability that the agent repeats the same action, regardless of whether rewarded. Agents that calculate mean reward using a moving average window = 1 trial exhibit more switching behavior, especially for non-rewarded trials.

Fig 7A and 7B summarizes the performance of 40 agents and show that the probability of the agent choosing *left* is determined by the probability of being rewarded for *left*, but also is influenced by the right reward probability, as previously reported [43]. The probability of a left response is similar for the agent with one Q matrix and the agent with G and N matrices (Fig 7B); however, the mean reward per trial is slightly higher for the agent with G and N matrices: 3.70 ±0.122 per trial for the agent with G and N and 3.43 ± 0.136 per trial for the one Q agent (T = -1.43, P = 0.155, N = 40). Agents require several blocks of 10 trials to learn the optimal side; however, they do change behavior after a single non-rewarded trial. Fig 7C shows that the probability of repeating the same response is lower after a non-rewarded (lose) trial. The probability of repeating the response is lower with a lower minimum value of β_1_ or a shorter window for calculating the mean reward.

**Fig 8:**
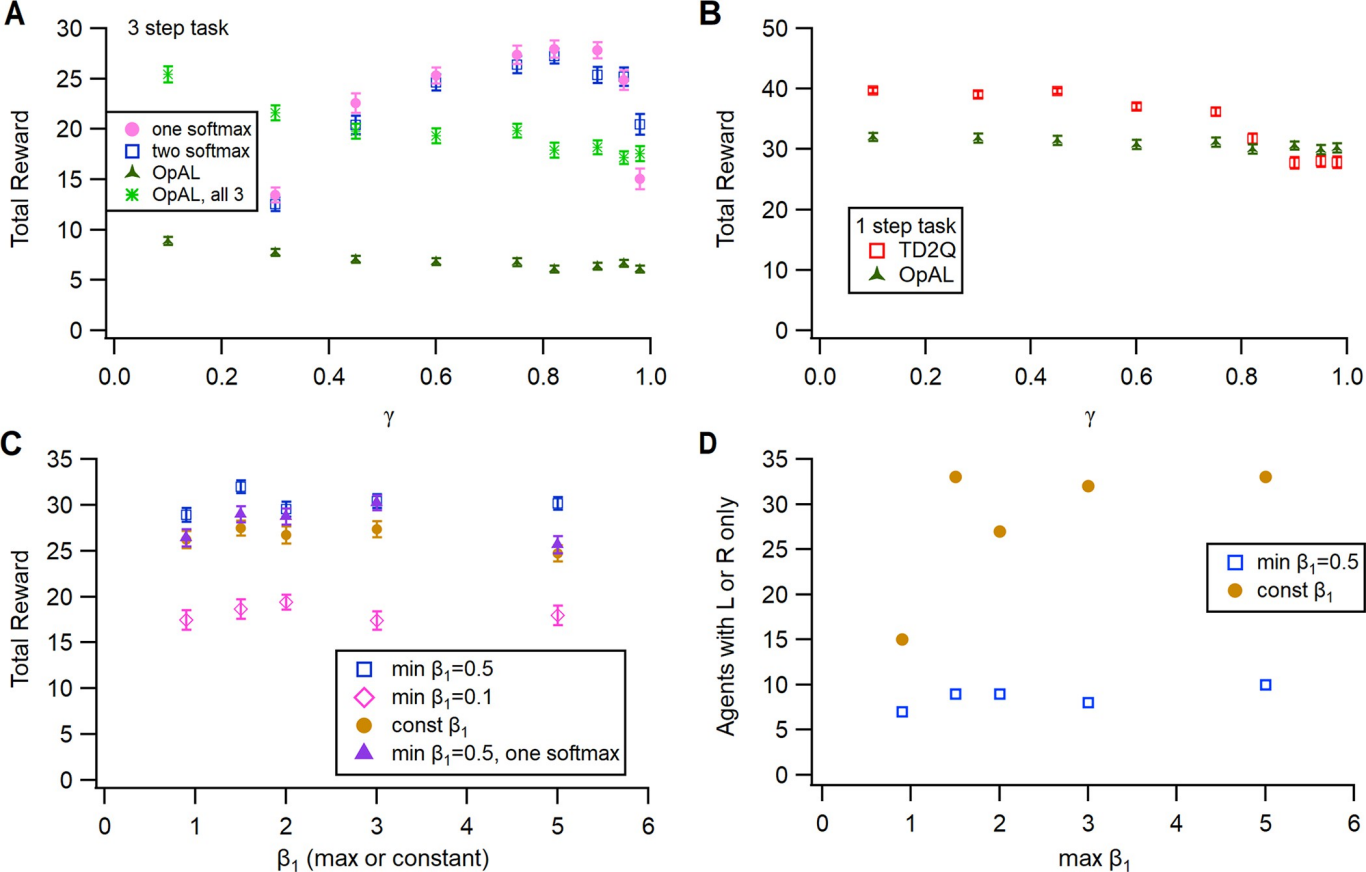
Exploitation-exploration parameter β_1_ and γ influence specific aspects of task performance. **A.** Total reward (reward per trial summed over all probability pairs) is sensitive to γ, though varies little with γ between 0.6 and 0.9. Using one softmax applied to the difference between G and N values had similar sensitivity to γ. **B.** The need for temporal difference learning is due to the number of steps in the task. A 1-step version of the task achieves optimal reward with γ between 0 and 0.6. **C.** Total reward has very low sensitivity to minimum and maximum value of β_1_. Using a constant value of β_1_ neither increases nor decreases total reward (F(4,394) = 2.52, P = 0.113). Using one softmax applied to the difference between G and N values had similar sensitivity to β_1_**. D.** Using a constant value of β_1_ reduces the likelihood that the agent samples both left and right actions when reward probabilities are the same for both actions (50:50). The symbols show the number of agents (out of 40) with only a single type of response (*left* or *right*).

Fig 8 summarizes the features required for the task performance. The temporal difference rule is critical, as decreasing γ toward 0 dramatically reduced the reward obtained (Fig 8A). If γ is greater than 0.9 or below 0.6, total reward declines, but within the range of 0.6–0.9, the performance is robust to variation in the value of γ. Note that the temporal difference rule is not required (γ can be 0) using a one-step version of the task (Fig 8B), i.e., with one state and two actions, and in each trial the agent selects *left* or *right* and receives reward or no reward. However, the 3 step task (go to center port, go to left or right port, and go to the reward site) with several possible irrelevant actions, better mimics rodent behavior during the task.

To further investigate the function of the temporal difference rule and the learning rules for updating Q values, we implemented the OpAL learning rule, which multiplies the change in Q value by the current Q value, uses a “critic” instead of the temporal difference rule, and initializes Q values to 1.0. Fig 8B shows that this version of OpAL learns a 1 step version of the task quite well, with optimal learning rates of α_G_ = α_N_ = 0.1. In contrast, this version of OpAL cannot learn the 3 step task, unless each step is rewarded (Fig 8A). Inspection of the Q values and (for OpAL) the critic values for one agent (90:10) reveals that they remain near zero for the initial state. S1 Fig revealed that the G values (used to calculate the RPE in TD2Q) are moderately high for action *center*, but that the critic value is negative for (*start*, *blip*). This negative critic value for OpAL prevents an increase in the G value for action *center*. The critic value is elevated for state (*poke port*, *6 kHz*), as are the G values for *Left* in this state; however the agent rarely reaches this state.

Performance on this task is sensitive to the minimum value of β_1_ and the moving average window for reward probability. The β_1_ value decreases when the reward rate drops following a change in reward probabilities, and increases to β_max_ when the agent has learned the side that provides 90% probability of reward (Fig 6D). If the moving average window is 1 trial, or β_1_ is too low (Fig 8C), then the agent is not sufficiently exploitative and receives fewer rewards. Using one softmax applied to the difference between G and N values produces similar rewards (Fig 8A and 8C). On the other hand, if the moving average window is too long or β_1_ is too high, then the agent is not exploratory and is impaired in switching responses when probabilities change (Fig 8D).

State-splitting is not essential for this task, as eliminating it does not change the mean rewards or probability matching. The agent learns the changing probabilities by changing G and N values dynamically, as probabilities change (Fig 6B and 6C). This is in contrast with latent learning models, in which the agent can learns a new latent state when the probabilities change. The reward is 3.70 ±0.122 per trial with state splitting, versus 4.03 ± 0.116 without state splitting.

### Sequence learning

**Fig 9:**
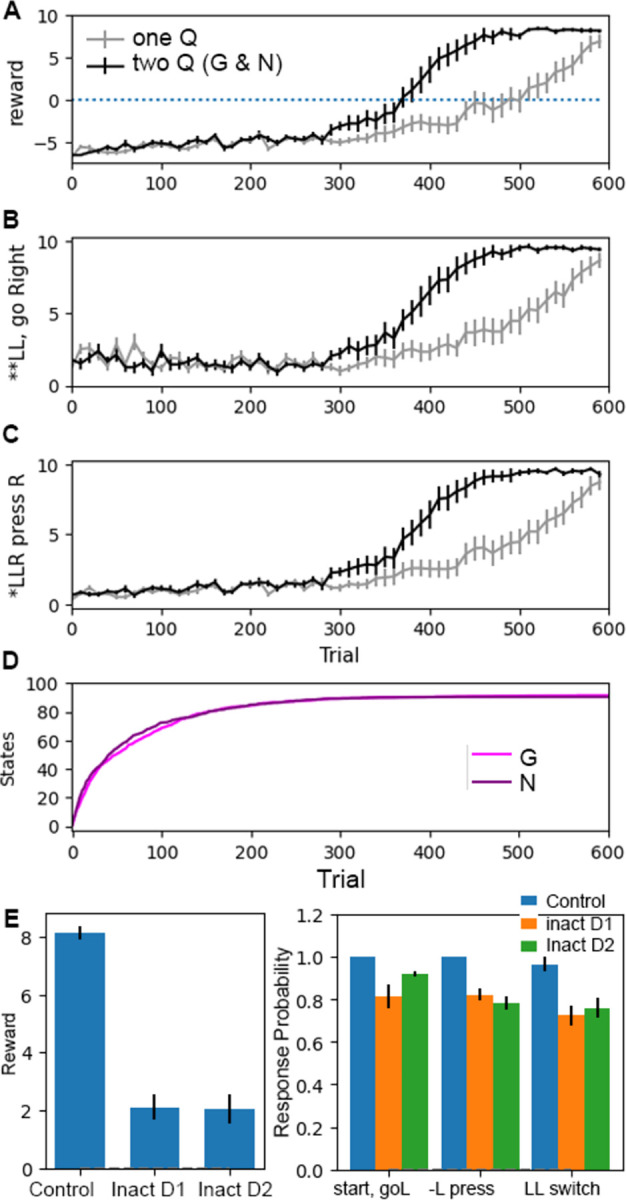
Faster learning in the sequence task with two Q matrices. **A**. Mean reward per trial increases sooner, though the final value does not differ between the agents with G and N matrices compared to one Q matrix (T = -1.8, P = 0.091, N = 15 each). **B.** Agent with G and N matrices learns to go to the right lever after two left presses beginning after 300 trials. **C.** Agent with G and N matrices learns to press right lever when press history is *LLR after 300 trials. B and C show number of events per block of 10 trials. In C, *LLR can be either *LLLR* or *RLLR*. Similarly, in B each * can be either *L* or *R*. **D.** The number of states in the G and N matrices increase during the first 100 trials, and then levels off as the agent learns the task. **E.** After training, inactivating G or N produces performance deficits. Effect of inactivation on reward: F(2,27) = 79.1, P = 5.73e-15. Post-hoc test shows that the difference between inactivating G and N is not significant (p = 0.91). Inactivation also reduced the correct start response (*start*, *goL*), correct press versus premature switching (*-L press*), and correct switching versus overstaying (*LL switch*).

We tested the TD2Q model in a difficult sequence learning task [44], in which the agent must press the left lever twice, and then the right lever twice (*LLRR* sequence) to obtain a reward. There are no external cues to indicate when the left lever or right lever needs to be pressed. Fig 9 shows that the agent with G and N matrices learned the task faster than an agent with one Q matrix. The difference in reward and responding at the end are not statistically significant (T = -1.8, P = 0.091, N = 15 each). The slow acquisition for this task (the agent with G and N requires ~400 trials to reach near optimal performance) is comparable to the 14 days of training required by mice [44]. The number of states (Fig 9D) are similar for agents with one Q versus G and N.

To test the role of G and N matrices in this task, we implemented the inactivation of G or N (setting values to 0) after training, similar to the experimental inactivation of D1-SPNs or D2-SPNs [44]. Inactivation of the G (N) matrix was accompanied by a bias applied to the G and N probabilities at the second decision stage to mimic the increase (decrease) in dopamine due to disrupting the feedback loop from striosome D1-SPNs to SNc. Fig 9E shows that inactivating either the G or N matrix (after learning) produced a performance deficit, as seen in the inactivation experiments [44]. We evaluated which aspects of the sequence execution were impaired by inactivation, and whether agents had difficulty initiating the sequence, switching prematurely to the right lever versus pressing a second time, or staying too long on the left lever versus switching after the second press. Fig 9E shows that the probability of a correct response was reduced for initiation (F(2,43) = 8.16, P = 0.001), second press on the left lever (F(2,43) = 26.43, P = 3.71e-8), and switching after the second left lever press (F(2,43) = 9.55, P = 0.00038). As observed experimentally [44], the impairment in initiation was more severe when the G matrix (corresponding to D1-SPNs) was inactivated, though this difference did not reach statistical significance (P = 0.068).

**Fig 10:**
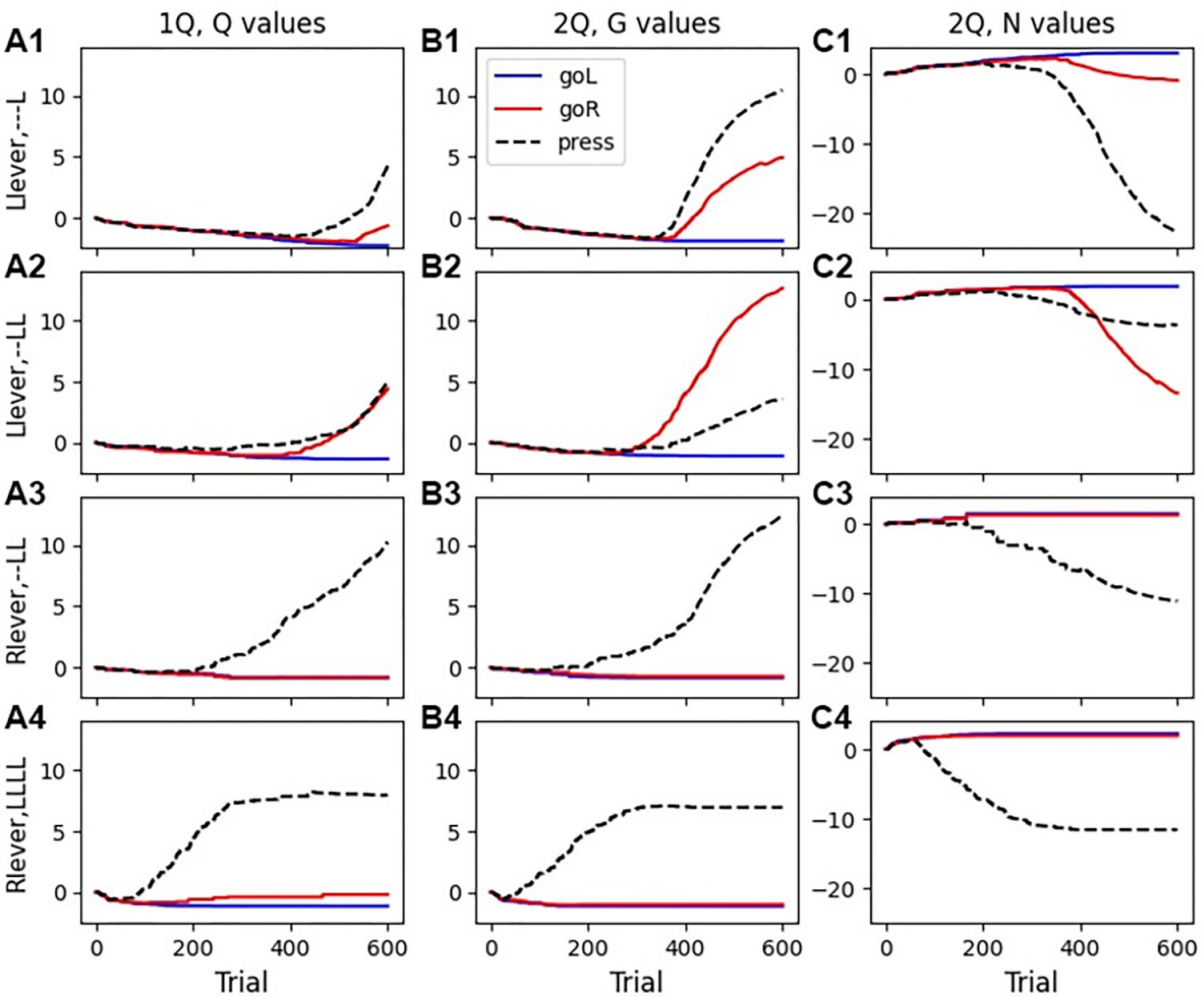
Q values for the sequence task for a subset of states. **A.** One Q agent. **B.** G values for agent, **C.** N values for agent. **Row 1:** When agent is at the left lever (*Llever*) and has only pressed once, the value for *press* is higher than the value for *goR*. **Row 2:** When agent is at the left lever (*Llever*) and has recently pressed the left lever twice, the G value for *goR* (switching) is higher than the G value for *press*, whereas the Q value for *press* and *goR* are similar for the one Q agent. **Rows 3–4:** When agent is at the right lever (*Rlever*), the Q value for *press* is high for agents with one Q as well as G and N, all other actions have Q values less than or equal to zero.

Fig 10 shows the state-action values for some of the states of this task. As the agent learns the task, the G value (Fig 10B2) increases and the N value (Fig 10C2) decreases for the action *go Right* for the state corresponding to (*Left lever*,--*LL*). The value for *go Right* for the one Q agent also increases (Fig 10A2), but so does the *press* Q value, which contributes to less than optimal performance. For the states corresponding to *Right lever*, the G value (Fig 10B3–10B4) is high and the N (Fig 10C3–10C4) value is low for action *press* when the two most recent presses are *left left*.

**Fig 11:**
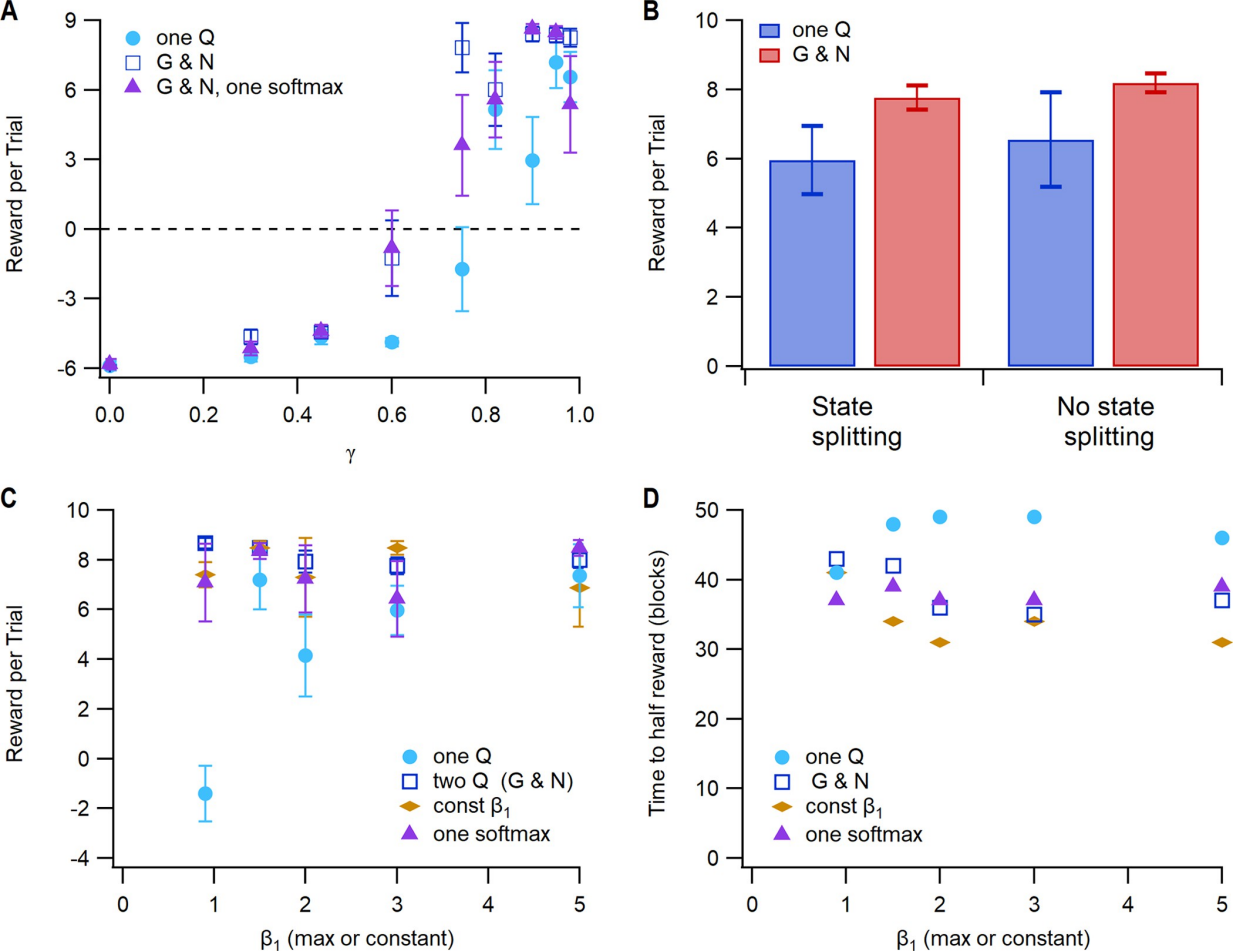
Exploitation-exploration parameter β_1_ and γ influence specific aspects of task performance. **A.** Reward per trial is highly sensitive to γ, especially for the one Q agent. Using one softmax applied to the difference between G and N values has similar sensitivity to γ, though is more variable. **B.** State splitting is not needed for this task and does not influence reward per trial. Both reward per trial **(C)** and Time to reach half reward **(D)** have very low sensitivity to minimum and maximum values of β_1_. The one Q agent has the lowest reward and slowest time to half reward. Using a constant value of β_1_ yields the fastest time to half reward.

Performance on this task is sensitive to γ and the limits of β_1_ (Fig 11). As observed with the previous two tasks, the temporal difference learning rule is critical, as reducing γ impairs performance (Fig 11A). In fact, this task requires a higher value of γ (0.95 versus 0.82), likely due to the larger number of steps required for reward and the need to more heavily weight future expected rewards. The one Q agent was more sensitive to the value of γ. This task also requires higher values of β_1_ than the other tasks (Fig 11C and 11D), because the agent does not need to be exploratory once it learns the correct behavior. Prior to learning, the agent is highly exploratory because none of the N or G values are high. Though reward rates are not higher using a constant β_1_, the time to reach the half reward rate is shorter (Fig 11D). Similar to the case with the serial reversal learning, sequence learning is neither helped nor impaired by state-splitting for either agents with one Q or G and N (Fig 11B) (F = 0.04, P = 0.84). We evaluated performance using the action selection rule used by OpAL–applying a softmax to the difference between G and N matrices. Fig 11A, 11C and 11D show that the agent can achieve a similar reward rate and time to reach the half reward rate, though it is more sensitive to the value of γ.

## Discussion

We present here a new Q learning type of temporal difference reinforcement learning model, TD2Q, which captures additional features of basal ganglia physiology to enhance learning of striatal dependent tasks and to better reproduce experimental observations. The TD2Q learning model has two Q matrices, termed G and N [26,53], representing both direct- and indirect-pathway spiny projection neurons. A novel and critical feature is that the learning rule for updating both G and N matrices uses the temporal difference reward prediction error (with the negative TD RPE used for the N matrix). The action is selected by applying the softmax equation separately to G and N matrices, and then using a second softmax to resolve action discrepancies. The TD2Q model also incorporates state splitting [14] and adaptive exploration based on average reward [34,35]. We test the model on several striatal dependent tasks, both cued operant tasks and self-paced instrumental tasks, each of which requires at least three actions to obtain a reward. We showed that using the temporal difference reward prediction error is required for multi-step tasks. Using two matrices allows us to demonstrate the role of indirect-pathway spiny projection neurons in discrimination learning. Specifically, blocking increases in N values, which is comparable to blocking LTP in indirect-pathway spiny projection neurons [19], impairs discrimination learning, but not acquisition, as observed experimentally. We also show that state-splitting is essential for renewal following extinction, and that using G and N matrices improves performance on a difficult sequence learning task.

Using the temporal difference reward prediction error (TD RPE) is critical for these multi-step tasks, as lower values of γ impairs performance. The optimal value of γ likely depends on the number of steps required for a task, as the optimal value of γ was higher for the 7 step sequence task compared to the 3 step tasks. Furthermore, γ = 0.1 or even 0 (i.e., not using the temporal difference rule) is sufficient when the switching reward probability task is simulated as a 1 step task. Though we only used the G matrix to calculate the TD RPE, using the N matrix to calculate its own TD RPE gave similar results. Future simulations will evaluate alternative calculations, such as TD RPE calculated as the difference between G and N [54], or using the difference between TD RPE calculated from G and TD RPE calculated from N. In summary, performance of TD2Q on multi-step tasks depends not only on the use of two Q matrices, but also using the temporal difference reward prediction error.

A key characteristic of TD2Q, the use of two Q matrices, is shared with several previously published actor-critic models. The OpAL model [26] has two sets of actor weights: one set for D1-SPNs and one set for D2-SPNs. OpAL’s learning rule differs in the use of the reward prediction error multiplied by the current G or N values. A second difference is that TD2Q uses the temporal difference between the value of the current state and the value of the previous state-action combination to calculate the reward prediction error, instead of a critic. A disadvantage of OpAL is that using a critic for the reward prediction error does not support learning multi-step tasks, unless each step is rewarded. Using the OpAL learning rule with the temporal difference reward prediction error does not yield good performance, either. Several models by Bogacz and colleagues also use two Q matrices [53,55]. One of the models [53] accounts for the effect of satiation on state-action values. One implementation of satiation has learning rules quite similar to TD2Q, except that the level of satiation determines the ratio of learning rates (α_G_ /α_N_). Another implementation reduces the learning rate for negative RPEs for G and for positive RPEs for N, and also includes a decay term. Neither implementation uses the temporal difference; thus, these models likely cannot learn multi-step tasks.

An advantage of the learning rules for both OpAL and models by Bogacz and colleagues [53,56] is that G and N values encode positive and negative prediction errors, respectively, and thus implement the observation that learning in response to punishment differs from learning in response to reward [57,58]. Another model to implement this observation is Max Pain [59], which has two Q matrices: one that maximizes the Q value in response to positive reward and another that minimizes the Q value in response to negative reward. The OTD model [60] also implements two Q matrices that learn positive and negative rewards and additionally has two different sets of inputs, corresponding to intratelencephalic and pyramidal tract neurons projecting to the D1- and D2-SPNs, respectively. The advantage of the OTD model is that it can account for calculation of the temporal difference reward prediction error by dopamine neurons; however, that model has not yet been evaluated on behavioral tasks.

Action selection in TD2Q differs from that in other two Q models. Several of the models [26,53] apply the softmax equation one time, to the (weighted) difference between the G and N weights. In Max Pain [59], the probability distribution for each action given the state is calculated using the softmax equation, and then the weighted sum of those probabilities is used to select the action. In contrast, TD2Q uses two levels of softmax equations. The first softmax selects an action for each Q matrix. Then a second softmax is applied to the probabilities associated with the 1^st^ level selected actions to determine the final action. We evaluated the performance of TD2Q when a single softmax was used for action selection, and found a subtle difference (Fig 11). However, an advantage of the two level softmax decision rule is that it is naturally extendable to even more biological Q learning models that have multiple, e.g. four (or more) Q matrices, representing D1- and D2-SPNs in both dorsomedial and dorsolateral striatum. Specifically, the dorsolateral striatum is more involved in habit learning [61–63], and some evidence suggests that synaptic plasticity in the dorsolateral striatum does not require dopamine [64]. Thus, the behavioral repertoire of Q learning models may be extended by adding two more Q matrices, representing D1- and D2-SPNs in dorsolateral striatum, with learning rules that depend more on repetition than reward (e.g. RLCK in [65]). A softmax for the second level of decision making then can be applied to the set of selected action probabilities of all four Q matrices.

The improvement in performance with two Q matrices on the sequence learning task leads to the experimental prediction that inactivation of D2-SPNs would delay learning of this task. Experimentally, inactivation of D2-SPNs increases rodent activity [22], but the effect on learning may depend on striatal subregion [66] or task [67]. Reproducing the experimentally observed performance deficit on the sequence task (inactivation applied after the mice had learned the task) required biasing the G and N probabilities at the second decision stage. The biological justification for this bias is based on recent research on striosomes, a subdivision of the striatum that is orthogonal to the D2-SPN - D1-SPN subdivision [68], revealing an asymmetry in the striatal control of dopamine release [31–33]. D1-SPNs in striosomes directly project to the dopamine neurons of SNc, which project back to the striatum. Thus, only the D1-SPNs directly influence dopamine release, though D2-SPNs indirectly influence dopamine release by inhibition of D1-SPNs. To mimic the increase in dopamine due to disrupting the feedback loop from striosome D1-SPNs to SNc (i.e., by inactivating D1-SPNs), the G values were increased, and to mimic the decrease in dopamine due to less D1-SPN inhibition by D2-SPNs (i.e., by inactivating D2-SPNs), the N values were increased. This leads to the prediction that experimental inactivation of D1-SPNs or D2-SPNs in the matrix only (avoiding the striosomes that control dopamine neuron firing) would not produce a performance deficit.

Another key feature in TD2Q is the dynamic creation of new states using state-splitting [14], which avoids the need to initialize Q matrices with values for all states that the agent may visit, and captures new associations during extinction in a different context. If the state cues are not sufficiently similar to an existing state, a new state is created. This is similar to the idea of expanding the representation of states if new states differ substantially from prior experience [69]. For example, if extinction is performed in a new context, a new state is created, and then reward prediction in that new state is extinguished. In addition, state splitting supports renewal: after the agent extinguishes in a novel context, it responds as before when returned to the original context. State splitting also results in generalization at the start of the discrimination trials: when presented with a 10 kHz tone, the agent responds *left*, as experimentally observed [19], because a new state splits from the *(center port*, *6 kHz)* state. The state splitting in TD2Q differs from the previous state-splitting in that the best matching state is determined using a Euclidean distance, though the Mahalonibis distance could also be used. In all cases tested, state splitting reduces the total number of states and thus reduces memory requirements, because the G and N matrices do not include states that are never visited. This feature is not critical for discrimination learning or the switching reward probability task, as many previous models can perform these tasks [11,13–16,26] and the total number of states is relatively small (less than 20). In contrast, the sequence task has numerous states, with 31 possible press histories and four locations; however, only ~90 of these states are instantiated during the task. A further reduction could be achieved by deleting (i.e., forgetting) rarely used states with low Q values. The biological correlate of state splitting can be refinement of the subset of SPN neurons that fire in response to cortical inputs. Each SPN receives highly convergent input from cortex [1,2,70], and some SPNs may receive similar subsets of cortical inputs and learn the same state-action contingency during acquisition. When introduced to a different context, a subset of those neurons may become more specialized by learning the new context, which would correspond to state-splitting. Note that the state values can differ between G and N matrices, which reduces the constraints on Q learning algorithms with multiple Q matrices. The different states are caused by the added noise or uncertainty about the input cues, and the slightly different state thresholds. Allowing different states reflects cortico-striatal anatomy–each SPN receives a different set of 10s to 100s of thousands of cortical inputs, and learns to respond to a different set of cortical inputs. Moreover, different cortical populations project to direct and indirect pathway SPNs, as pointed out by [54].

State splitting, especially TDRLXT, has some similarity to latent state learning in that both learn two types of information–(1) which cues are relevant to determine the correct state (or context) and (2) what is the associative value (or state-action value) of the cues–and can add new states (or latent states) as needed; however, there are key differences. TDRLXT [14] uses mutual information to determine the relevance of both context and cues. In addition, both TD2Q and TDRLXT determine whether to create a new state by evaluating similarity to previously learned states. In contrast, latent state learning [46,69,71,72] uses Bayesian inference to determine both the associative strength of the cue as well as how to treat contextual cues–e.g., as additive (i.e., another cue) or modulatory (i.e., changing the contingencies). In both types of models, temporal contiguity or novelty can be used to determine the state or latent state [14,72], though TD2Q uses novelty only. TD2Q could be improved further by implementing methods used by humans and animals to identify relevant cues, such as using mutual information as in [14] or clustering as in [72].

Action selection in both reinforcement learning and latent learning models is controlled by a free parameter, called the exploitation-exploration parameter, β_1_, in the softmax equation. Reward-based control of β_1_ is critical for tasks in which the best action changes periodically, i.e., the switching reward probability task. Thus, both this task and reversal after discrimination needed lower values of β_1_ than the sequence task. In our model, the variation of β_1_ between β_min_ and β_max_ depends on the average reward obtained for the previous few trials [34]. This is analogous to dopamine control of action selection, in which an increase in exploratory behavior after several trials without reward is observed experimentally [38,73]. When reward probabilities shift, the TD2Q agent obtains less reward and becomes more exploratory, without explicitly recognizing a new context or latent state [46]. Exploration also may be controlled by uncertainty about reward, e.g., variance in reward [37,74–77]. Several methods have been proposed for accounting for the reward variance [78], which can enhance or reduce exploration. In the switching reward probability task herein, the uncertainty (probability) of reward is correlated with mean reward; thus whether mean or variance of the reward is driving exploration cannot be determined. Exploitation versus exploration also can be controlled by scaling β_1_ by the variance in Q values for that state or when the context changes [79].

What part of the brain controls exploitation versus exploration? The internal segment of the globus pallidus (GPi, entopeduncular nucleus in rodents) and substantia nigra pars reticulate (SNr) are sites of convergence of direct and indirect pathways [80,81], making these likely sites for decision making. The GPi also receives dopamine inputs that shift how GPi neurons respond to indirect versus direct pathway inputs [81,82]. Thus, we predict that blocking dopamine in the GPi and SNr would impair probability matching in the switching reward probability learning task. Decision making also may be controlled in the striatum itself, by inhibitory synaptic inputs from other SPNs [83,84] or interneurons, which also undergo dopamine dependent synaptic plasticity [85–88]. Previous studies suggest a role of noradrenaline in regulating exploration [89–91]. Noradrenergic inputs to the neocortex may influence decision making through control of subthalamic nucleus interactions with the globus pallidus.

Though TD2Q and other reinforcement learning models correspond to basal ganglia circuitry, research clearly shows the involvement of other brains regions in many striatal dependent tasks. Numerous studies have shown the importance of various regions of prefrontal cortex for goal-directed learning [92,93]. For example, switching reward probability learning is impaired by prefrontal cortex lesions [94–96]. Processing of context, such as spatial environment, is performed by the hippocampus [97–100]. Thus, one class of reinforcement learning models allows the agent to create an internal model of the environment [101–104]. These models are particularly adept in spatial navigation tasks, although with significantly greater computational complexity. Given that hippocampus and prefrontal cortex provide input to the striatum, a challenge is to use models of learning, planning and spatial functions of these regions as inputs to striatal based reinforcement learning models.

As one of our goals is to improve correspondence to the striatum and to understand the role of different cell types and striatal sub-regions, future models should allow Q matrices to represent synaptic weight and post-synaptic activation as distinct components, as in [26] or where Q matrices are learned for each component of a binary input vector. Using this latter approach, the Q values for each vector component represents synaptic weights and the total Q value represents post-synaptic activity [45,71,72,105]. Future models also should implement additional Q matrices to represent dorsomedial and dorsolateral striatum [106]. Numerous behavioral experiments have shown that dorsomedial striatum promotes goal-directed behavior, whereas dorsolateral striatum promotes habitual behavior. Action selection with additional Q matrices arranged in parallel or hierarchically is a possible extension to the current action selection [11,107].

## Supporting information

S1 Fig G values and critic values for agents performing the 3-step switching reward probability task. Probability of reward was 90% for action *left* in state *(poke port*, *6 kHz*) and 10% for action *right*. **A.** Critic value for two of the states for the agent implementing the OpAL learning rule. **B**. G values for actions when agent is in the state (*start*,*blip*). OpAL agent does not learn the action *center*. **C.** G values for actions when agent is in the state (*poke port*, *6 kHz*). Both agents learn the best action *left*.
(TIF)
