# Supplementary figures and images for: Enhancing reinforcement learning models by including direct and indirect pathways improves performance on striatal dependent tasks
Source: PLoS Comput Biol. 2023 Aug 18;19(8):e1011385. doi: 10.1371/journal.pcbi.1011385 (PMC10479916; doi:10.1371/journal.pcbi.1011385)

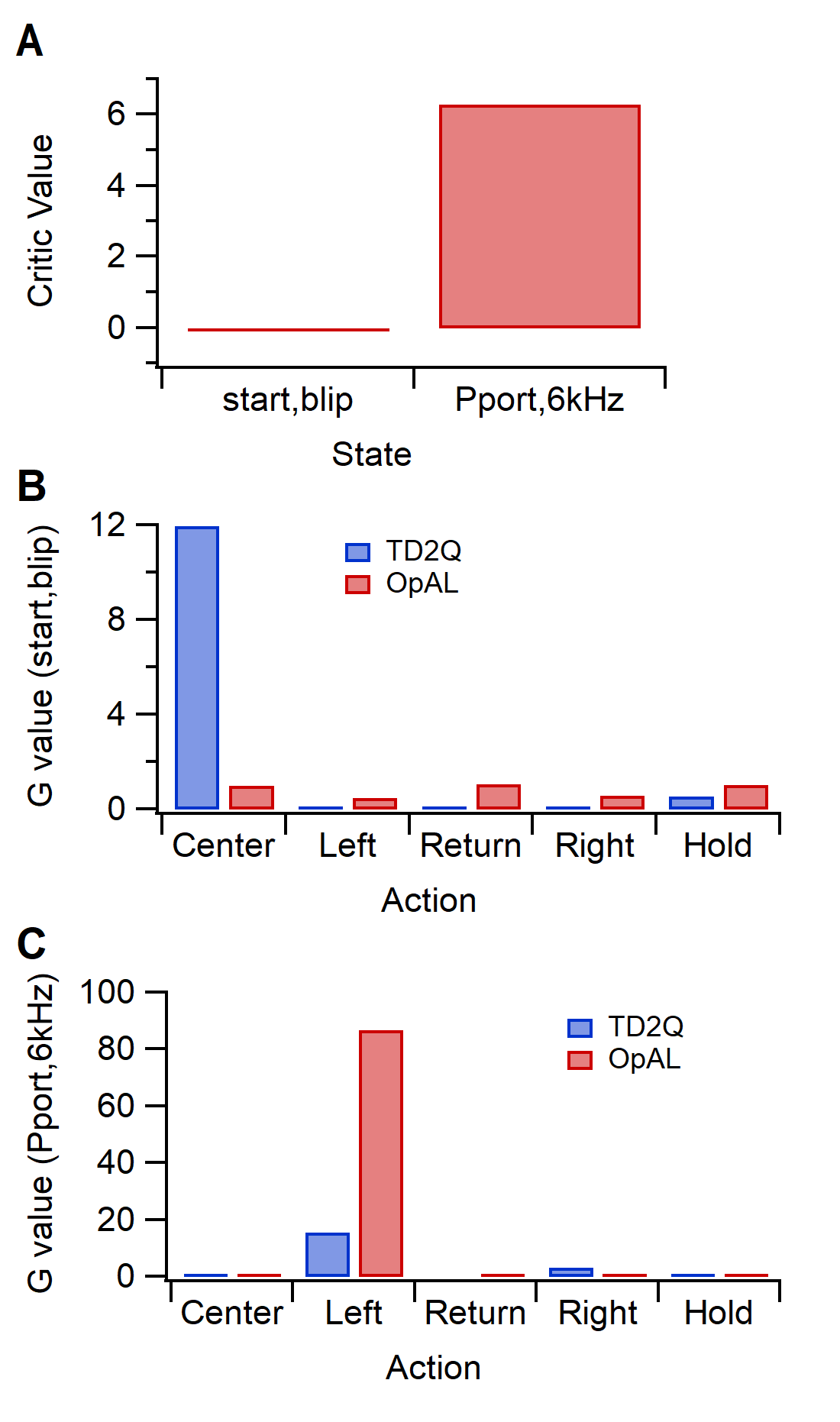

Supplement: S1 Fig — Probability of reward was 90% for action left in state (poke port, 6 kHz) and 10% for action right. A. Critic value for two of the states for the agent implementing the OpAL learning rule. B. G values for actions when agent is in the state (start,blip). OpAL agent does not learn the action center. C. G values for actions when agent is in the state (poke port, 6 kHz). Both agents learn the best action left. (TIF) [file pcbi.1011385.s001.tif]
